# Virus-Mediated Chemical Changes in Rice Plants Impact the Relationship between Non-Vector Planthopper *Nilaparvata lugens* Stål and Its Egg Parasitoid *Anagrus nilaparvatae* Pang et Wang

**DOI:** 10.1371/journal.pone.0105373

**Published:** 2014-08-20

**Authors:** Xiaochan He, Hongxing Xu, Guanchun Gao, Xiaojun Zhou, Xusong Zheng, Yujian Sun, Yajun Yang, Junce Tian, Zhongxian Lu

**Affiliations:** 1 State Key Laboratory Breeding Base for Zhejiang Sustainable Pest and Disease Control, Institute of Plant Protection and Microbiology, Zhejiang Academy of Agriculture Sciences, Hangzhou, China; 2 Jinhua Research Academy of Agricultural Sciences, Jinhua, China; 3 School of Medicine Science, Jiaxing University, Jiaxing, China; Institute of Vegetables and Flowers, Chinese Academy of Agricultural Science, China

## Abstract

In order to clarify the impacts of southern rice black-streaked dwarf virus (SRBSDV) infection on rice plants, rice planthoppers and natural enemies, differences in nutrients and volatile secondary metabolites between infected and healthy rice plants were examined. Furthermore, the impacts of virus-mediated changes in plants on the population growth of non-vector brown planthopper (BPH), *Nilaparvata lugens*, and the selectivity and parasitic capability of planthopper egg parasitoid *Anagrus nilaparvatae* were studied. The results showed that rice plants had no significant changes in amino acid and soluble sugar contents after SRBSDV infection, and SRBSDV-infected plants had no significant effect on population growth of non-vector BPH. *A. nilaparvatae* preferred BPH eggs both in infected and healthy rice plants, and tended to parasitize eggs on infected plants, but it had no significant preference for infected plants or healthy plants. GC-MS analysis showed that tridecylic aldehyde occurred only in rice plants infected with SRBSDV, whereas octanal, undecane, methyl salicylate and hexadecane occurred only in healthy rice plants. However, in tests of behavioral responses to these five volatile substances using a Y-tube olfactometer, *A. nilaparvatae* did not show obvious selectivity between single volatile substances at different concentrations and liquid paraffin in the control group. The parasitic capability of *A. nilaparvatae* did not differ between SRBSDV-infected plants and healthy plant seedlings. The results suggested that SRBSDV-infected plants have no significant impacts on the non-vector planthopper and its egg parasitoid, *A. nilaparvatae*.

## Introduction

The multi-trophic relationship involving plants, herbivorous insects, and natural enemies is the most basic component of nearly all ecosystems. Any change in the factors affecting plant growth can change the interactive relationships among the three trophic levels through a variety of mechanisms [1]. Plant viruses transmitted by arthropods are an important biological factor in agro-ecosystems. Viruses can affect not only the yield and quality of host plants, but also the growth, physiological and biochemical changes as well as the ecological characteristics of arthropods serving as the vector. Furthermore, they can have direct or indirect effects on non-vector herbivorous arthropods and their natural enemies, potentially impacting entire agro-ecosystems [2]–[4].

Viral infection in plants can cause changes in nutrient components of host plants. After being infected with rice black-streaked dwarf virus (RBSDV), diseased rice plants had a 31.13% increase in free amino acid levels, and soluble sugar content was three times higher than that in healthy rice plants [5]. The total amino acid content in the phloem of wheat (*Triticum aestivum*) declined after infection with barley yellow dwarf virus (BYDV) [6]. Plant secondary compounds are key information factors linking trophic relationships among plants, pests and natural enemies [7], [8]. After infection, the type and content of volatile substances in host plants changed, which in turn affected the behaviors of herbivorous arthropods and their natural enemies [9]. Mexican bean beetles (*Epilachna varivestis*) tended to choose those tissues infected with southern bean mosaic virus (SBMV) or bean pod mottle virus (BPMV), which were both caused by plant secondary compounds under viral infection [10]. Squash (*Cucurbita pepo*), infected with cucumber mosaic virus (CMV), exhibited a significant increase in the content of volatile secondary metabolites, and its attraction to aphis (*Myzus persicae*) and its parasitoid (*Aphid gossypii*) was strongly enhanced [11]. Many studies have focused on the interaction between viruses and host plants [6], [12], [13], and between viruses and vector insects [10], [14]–[16]. However, only a small number of studies have included the three trophic levels, consisting of viruses, insects (especially non-vector insects) and natural enemies [11].

The southern rice black-streaked dwarf virus (SRBSDV) was first discovered in Guangdong China in 2001 as a new rice virus transmitted by rice whitebacked planthopper (WBPH), *Sogatella furcifera* (Horváth) [17], [18]. In 2011 and 2012, its total distribution areas in China and Vietnam were 700,000 and 500,000 hectares, respectively [19], [20]. Currently, the virus is also found in Japan [21]. It was reported that the viruliferous WBPH laid significantly fewer eggs than non-viruliferous hoppers. There were no significant differences in the hatchability of eggs laid by virulifierous and non viruliferous females [22]. This study using paired viruliferous and nonviruliferous WBPH showed that both infected females and males had significantly reduced fecundity and F1 egg hatchability. When paired with either a non viruliferous female or male, there were no significant effects in fecundity and egg hatchability [23]. In paddy fields, the brown planthopper (BPH), *Nilaparvata lugens*, normally coexists with WBPH and always shares its host rice plants with WBPH in east China [24], [25]; however, BPH is not the vector of SRBSDV. To further understand the ecological impacts of the plant virus, this study investigated the effects of the changes in nutrients and volatile secondary metabolites of host plants after SRBSDV infection on the population growth of non-vector brown planthopper (BPH), *Nilaparvata lugens*, as well as the selectivity and parasitic capability of its egg parasitoid *Anagrus nilaparvatae*. We sought to clarify the wider ecological role of the plant virus in each trophic level of rice plants and to provide a solid basis for better prevention and sustainable management of rice planthoppers during SRBSDV epidemics.

## Materials and Methods

### Rice plants

The rice variety for rearing BPH and WBPH, susceptible TN1, was provided by the International Rice Research Institute (IRRI), the Philippines. After indoor seed germination, seedlings were planted in a cement sink in a netted room free of insects. At 3-leaf stage, seedlings were transplanted into pots (diameter 9 cm) in the netted room. 45–60 d old plants were fed to BPH and WBPH.

Rice variety Y-Liangyou 1 was used in experiment. It is a rice variety susceptible to SRBSDV and the dominant *indica* hybrid rice in Zhejiang province, China.

### Rice planthoppers

Vector WBPH and non-vector BPH were collected from paddy fields of the China National Rice Research Institute (CNRRI), Hangzhou (119.95°E, 30.07°N). Dr. Fu Qiang (fuqiang@caas.cn) of CNRRI should be contacted for future permissions and there are no endangered or protected species involved. The planthoppers were maintained continuously on susceptible rice TN1 in an artificial climate chamber under the conditions of 26±1°C, 70–90% relative humidity, and L12:D12.

### Parasitoid

The egg parasitoid of rice planthoppers, *A. nilaparvatae* was trapped in the rice fields and bred continuously indoors. Gravid BPH female adults were caged for oviposition on potted rice plants for 24 h. After removal of the cage and BPH, the potted plants with BPH eggs were transferred to paddy fields in the experimental farm of the Zhejiang Academy of Agricultural Sciences, Hangzhou (120.18° E, 30.27° N). After exposing them in thefield for 48 h, plants with parasitized eggs were collected and returned to the artificial climate chamber under the conditions of 26±1°C, 70–90% relative humidity, and L12:D12. Each potted rice plant was covered with a polyethylene cage (height 60 cm, diameter 9 cm) and an outer black cloth. Its top had an opening of 1 cm in diameter and was connected to an inverted transparent glass tube (diameter 1 cm, length 6 cm). After the emergence of *A. nilaparvatae*, the glass tubes were replaced daily. In addition, after sexual identification, *A. nilaparvatae* in the tubes were placed into cages containing rice plants with fresh BPH eggs in several batches. The next generation of *A. nilaparvatae* was used for testing.

### Infected and healthy rice plants

Rice variety Y-Liangyou 1 was used in this experiment. It is a rice variety susceptible to SRBSDV and the dominant *indica* hybrid rice in Wuyi county (119.81°E, 28.9°N), Zhejiang province, China. The area has been subject to frequent SRBSDV outbreaks in recent years [26]. To obtain SRBSDV-infected plants, 2nd instar nymphs of WBPH were placed in a beaker padded with wet filter paper to make them hungry. After 2 h without feeding, they were transferred to SRBSDV-infected plants (Y-Liangyou 1) collected from a paddy field in Wuyi county for 2–3 d. They were then transferred and bred on healthy TN1 seedlings at the tillering stage for a circulative period of one week. Lastly, they were inoculated to Y-Liangyou 1 plants at 3-leaf stage [27], [28]. After the appearance of the typical SRBSDV-infected symptoms on the rice plants about 40 d old, the plants were marked and the leaves were individually sampled and molecularly identified by the methods of Li et al. (2012) [29]. 45–60 d old healthy and infected plants were used for testing.

### Chemicals used in the experiment

Standard samples of volatiles, including octanal (purity≥99%), methyl salicylate (analytical standard), undecane (analytical standard), hexadecane (analytical standard), and tridecanal (purity≥95%) were purchased from the Sigma-Aldrich Corporation. Using liquid paraffin as a solvent, solutions diluted 10^2^, 10^4^ and 10^6^ times were prepared for testing. Internal standards n-octane and nonyl acetate were purchased from Sigma-Aldrich; 400 ng of octane and 400 ng of nonyl acetate were weighed and mixed with 20 µL hexane as the internal standard.

### Determination of amino acid content in rice plants

Leaf sheaths of SRBSDV-infected 60 d old rice plants and corresponding healthy rice plants were sampled after RT-PCR determination. After de-enzyming for 1 h at 110 °C, they were dried at 80 °C to constant weight. One gram dried leaf was ground into powder, and 0.1% HCl was added to the volume of 25 ml. The solution was filtered after complete dissolution; 2 ml of supernatant was mixed with 4 ml 0.1% TFA solution by rapid shaking. After purification with a SEP-PAK column, the solution was loaded onto an amino acid analyzer (Sykam S433D) for determination of amino acid content.

### Determination of soluble sugar content in rice plants

Soluble sugar content in rice plants was determined following the methods of Wang et al. (2010). Leaf sheaths from SRBSDV-infected plants and from corresponding healthy rice plants were sampled and dried to constant weight and ground into powder. About 0.1 g dried powder was placed into a large test tube, into which 15 ml of distilled water was added. After 20 min of boiling in a water bath, the sample was cooled and filtered into a 100 ml volumetric flask. The residue was washed with distilled water several times to constant volume; 1.0 ml of sample extract was added to 5 ml of anthrone reagent. After rapid shaking and mixing of each tube, they were heated in a boiling water bath for 10 min, and cooled afterwards for measurement of OD_620_. AR anhydrous glucose was plotted as the standard curve to calculate soluble sugar content of the sample.

### Extraction and analysis of plant volatiles

Instruments and materials included a GCMS-QP2010 gas chromatograph - mass spectrometer (Shimadzu Corporation), a solid phase micro extraction (SPME) device (Supelco Inc. USA), an extraction head (Polyacrylate (PA) 85 µm, polydimethylsiloxane (PDMS) 100 µm, polydimethylsiloxane/divinybenzene (PDMS/DVB) 65 µm), and a capillary column Rtx-5MS (0.25 µm×30.0 m×0.25 mm).

Plants infected with SRBSDV and healthy plants of the same age were put into the adsorption device. The roots were wrapped with freshness-preserving film to prevent dirt and other air flow from mixing with the rice volatiles. Air from a blower was purified by passing it through distilled water and activated carbon. Afterwards, it entered a glass cylinder (diameter 10 cm, height 50 cm) from the top at 800 ml/min. After passing through the entire cylinder, it entered an adsorption column from the lower side at 600 ml/min. The inflowing air was greater than the outflowing air, which ensured that the air filled the entire glass cylinder. The adsorption column was connected to a pump, and the volatiles were continuously collected for 4 h (10:00–14:00).

After 4 h of collection, the adsorption column was removed and rinsed with 800 µL hexane. The eluent was put into a 1500 µL storage vial and added to 10 µL of internal standard (200 ng n-octane and 200 ng nonyl acetate were added to 20 µL n-hexane). The measurement was made after mixing evenly.

A micro-injector was used to load 1 µL of sample; a gas chromatograph - mass spectrometry (GC-MS) was used for analysis. Tests were conducted at 26±1°C and 60% relative humidity.

The components were analyzed qualitatively based on the degree of agreement between the mass spectrum of components in the sample and the spectrum generated by GC-MS ChemStation software as well as the degree of agreement between the retention time of the components in the sample and that of the standard compounds in GC-MS. Relative quantification was performed based on the area ratio of component peaks in the sample to the internal standard peak.

For chromatography, a capillary column (Rtx-5MS; 30 m×0.25 mm×0.25 µm) was used with carrier gas helium at 24 cm/sec (99.999%) and an initial column temperature of 40°C, which was maintained for 3 min, then increased to 230°C at 8°C/min, and maintained for 9.5 min. Splitless injection was performed.

For mass spectrometry an EI ion source was used with an ionization energy of 70 eV, an ion source temperature of 200°C, an inlet temperature of 250°C, and a mass scan range of m/z 45∼500. A library search was conducted using NIST08.L and NIST08s.L.

### Population growth of non-vector BPH on SRBSDV-infected and healthy plants

SRBSDV-infected plants and healthy rice plants were uprooted and separated into single plants. The outer sheathes and the inactive roots were removed. Afterwards, they were rinsed with tap water and placed in tubes (diameter 1.5 cm, height 15.0 cm) containing 1.5 cm deep Kimura B nutrient solution. Ten newly hatched nymphs within 24 h of BPH were inoculated into each tube, which were then sealed with degreased cotton. This procedure was repeated ten times for infected and healthy plants, respectively. Their growth and development was observed daily. The rice plants were replaced as needed until planthopper emergence. Within 12 h after adult emergence, a pair of BPH adults were inoculated into tubes containing either healthy or infected plants. They were then put into a biochemical incubator under conditions of 26±1 °C and 12 L: 12D, where they were allowed to mate and lay eggs. When the nymphs hatched, their numbers were counted each day and these nymphs were removed until no more nymphs hatched for 5 consecutive days. Afterwards, rice plants were dissected under a microscope. The number of unhatched eggs was recorded. Based on the number of hatched nymphs and the number of unhatched eggs, the hatching rate of eggs was calculated.

### Host selectivity of *A. nilaparvatae* for BPH eggs in SRBSDV-infected and healthy plants

A single SRBSDV-infected plant and a healthy rice plant of the same age were transferred into the same clay pot covered with a cage. After mating, three gravid BPH female adults for each plant were introduced into the cages for 2 d. After the BPH were removed, two pairs of newly emerged *A. nilaparvatae* were inoculated, and the parasitoids were removed after 24 h. After 5 d, the numbers of parasitized eggs and healthy eggs were counted by dissecting plants under a microscope. This experiment was conducted inan artificial climate chamber under the conditions of 26±1 °C, 70–90% relative humidity, and L12:D12. Replications were conducted ten times for each treatment.

### Preference of *A. nilaparvatae* for odor sources from SRBSDV-infected and healthy plants

The behavioral responses of *A. nilaparvatae* to different odor sources (as described in “Extraction and analysis of plant volatiles”) from SRBSDV-infected and healthy plants containing BPH eggs were measured using a Y-tube olfactometer. Lengths of the two arms and the straight tube of the Y-tube olfactometer were 10.0 cm, the inner diameter was 1.0 cm, and the inclusion angle between the two arms was 75°. An open ampoule holding 1 ml of odor source solution was placed inside the odor source bottle, and another opening ampoule holding 1 ml of liquid paraffin was placed in the control bottle. The two arms of the Y tube were in turn connected to the odor source bottle (or the control bottle), humidification bottle, air filter (with activated carbon) and flow meter by a Teflon tube, and the base of the Y tube was connected to a pump for air injection. During the measurement of behavior, air was pumped from the base of the Y tube. The flow rate of air in the two arms was regulated at 150 ml/min. When the Y tube was filled with a volatile odor source, one newly emerged *A. nilaparvatae* was introduced into the Y tube through its base port. Each *A. nilaparvatae* that entered one arm of the Y tube and moved upwind more than 5 cm was counted; otherwise it was not counted if there was no response after more than 10 min. For each odor source at each concentration level, 60 *A. nilaparvatae* were measured. For every 10 insects measured, anhydrous ethanol was used to wash and dry the tube. Afterwards, the two arms were swapped, and the connecting positions at the odor source bottles as well as CK bottles were adjusted to eliminate the possible effects of slight geometric differences in the two arms on the behavior of *A. nilaparvatae*. After finishing the determination with each concentration of each odor source, the Y tube and odor source bottles were washed, and then dried in an oven at 120 °C. The activated carbon for air filtering was heated in an oven at 100 °C for later use. Three treatment groups were set up: 1) healthy plants and clean air as control, 2) SRBSDV-infected plants and clean air as a control, and 3) SRBSDV-infected plants and healthy plants.

### Behavioral responses of *A. nilaparvatae* to single substances at different concentrations

Based on the results of the analysis of plant volatiles from infected and healthy rice plants, octanal, methyl salicylate, undecane and hexadecane were not detected from the SRBSDV-infected rice plants, and tridecanal was not found from healthy rice plant. In order to clarify the functions of those five chemical substances, behavioral responses of *A. nilaparvatae* to single substances at different concentrations were determined by using a Y-tube olfactometer. The method was described in detail above.

### Parasitic capability of *A. nilaparvatae* on BPH eggs in SRBSDV-infected and healthy plants

SRBSDV-infected plants and healthy rice plants were uprooted and separated into single plants. The outer sheaths and the inactive roots were removed. Afterwards, they were rinsed with tap water and individually placed into tubes (diameter 1.5 cm, height 15.0 cm) containing 1.5 cm deep Kimura B nutrient solution, which were then sealed with degreased cotton. Three gravid female BPH were introduced into each tube, and 24 h later, one female parasitoid emerging within 4 h was then inoculated into the tube. The tubes were kept in the artificial climate chamber under the conditions of 26±1 °C, 70–90% relative humidity, and L12:D12. Rice plants were dissected under a microscope on the 6th d after *A. nilaparvatae* died. Parasitic capability of *A. nilaparvatae* was measured by the number of parasitic eggs. Twenty-two replications were conducted for each treatment.

### Statistics and analysis

Isolation and identification of volatile compounds released by the rice plants were performed using the NIST08 mass spectral library. BPH population growth rate was calculated as follows:

Population growth rate  =  nymph survival × ratio of female adult × number of eggs laid by each female × egg hatching rate.

SPSS18.0 was used for independent samples t tests. A binomial distribution was used to test preference for different odor sources. Descriptive statistics were expressed as the mean ± standard error, and the significance level was set at α = 0.05.

## Results

### Changes in amino acid and soluble sugar contents in rice plants after SRBSDV infection

After SRBSDV infection, the content of various amino acids and the total amino acid content in the rice plants did not change significantly (t = −0.144, df = 6, P = 0.899) (Table 1). Soluble sugar content in the SRBSDV-infected plants was 6.69%, whereas the content in healthy plants was 6.00%. The difference was not significant (t = −1.060, df = 6, P = 0.330).

**Table 1:** Amino acid and soluble sugar in infected and healthy rice plants.

Amino acids	Healthy plants (% dry weight)	Infected plants (% dry weight)	Change rate (%)	*P*
Asp	2.24±0.04	3.04±0.10	35.71	0.050
Thr	0.88±0.02	0.85±0.03	3.98	0.393
Ser	0.86±0.02	0.87±0.03	1.75	0.712
Glu	2.33±0.05	2.40±0.08	3.23	0.519
Pro	0.88±0.02	0.86±0.03	1.71	0.712
Gly	1.00±0.02	0.91±0.03	8.54	0.170
Ala	1.22±0.02	1.09±0.04	11.07	0.107
Val	1.02±0.02	0.95±0.02	6.86	0.132
Met	0.18±0.00	0.17±0.01	5.56	0.423
Ile	0.80±0.02	0.72±0.03	10.06	0.137
Leu	1.64±0.03	1.42±0.05	13.46	0.077
Tyr	0.47±0.01	0.46±0.02	3.19	0.504
Phe	1.02±0.02	0.89±0.03	12.75	0.084
His	0.66±0.01	0.65±0.03	2.27	0.657
Lys	1.12±0.02	0.99±0.03	11.61	0.084
Arg	0.94±0.02	0.90±0.03	4.26	0.397
Total amino acids	17.23±0.28	17.14±0.56	0.52	0.899
Soluble sugar	6.00±0.35	6.69±0.56	11.50	0.330

### Plant volatiles from SRBSDV-infected and healthy rice plants

The results shown in Table 2 indicated that total 24 types of substances were identified as volatile components released by rice plants, including 23 types in healthy rice plants and 20 types in SRBSDV-infected plants. They were mainly composed of aldehydes, alcohols, alkanes, and lipids. Tridecylic aldehyde was collected only from SRBSDV-infected plants, whereas octanal, undecane methyl salicylate and hexadecane were only from healthy rice plants. The relative contents of methyl benzene, (Z)-3-hexenal, (E)-2-hexenal, 3-hexanol, 1-hexanol, heptyl aldehyde, 2-ethyl hexanol and nonyl aldehyde were elevated after SRBSDV infection. The contents of methyl benzene, (Z)-3-hexenal, (E)-2-hexenal, and 3-hexanol were significantly different (P<0.05).

**Table 2:** Comparison of volatile compounds emitted from healthy and SRBSDV-infected plants.

Chemicals	Healthy plants (ng/g)	SRBSDV-infected plants (ng/g)	*P*
methyl benzene	8.40±1.08	17.38±2.86	0.013
(Z)-3-hexenal	5.65±1.42	10.22±0.90	0.013
(E)-2-hexenal	7.54±2.19	12.91±3.26	0.035
3-hexanol	3.68±0.89	6.27±0.62	0.027
ethylbenzene	5.43±1.50	6.56±1.33	0.576
1-hexanol	1.08±0.23	1.24±0.11	0.539
heptanal	2.64±0.67	3.28±0.59	0.483
octanal	4.33±1.09	/	
2-ethyl hexanol	13.58±3.50	24.05±6.80	0.203
undecane (H)	7.92±2.28	/	
nonanal	7.95±2.45	8.72±1.77	0.799
menthol	3.00±1.27	2.07±0.66	0.510
naphthalene	4.19±1.02	3.89±0.99	0.839
methyl salicylate	1.04±0.78	/	
dodecane	2.18±0.60	1.70±0.51	0.548
decanal	5.39±1.60	4.13±0.95	0.498
dodecanal	4.45±1.62	4.48±0.95	0.985
(+)-longifolene	12.82±3.72	7.50±2.21	0.240
α-cedrene	21.25±6.38	8.13±2.61	0.084
β-caryophyllen+β-cedrene	10.24±3.13	3.15±0.91	0.056
dimethyl phthalate	112.35±32.98	94.73±38.36	0.735
hexadecane	26.72±7.87	/	
tetradecanal	4.72±1.87	3.51±0.71	0.538
α-cedrol	9.73±2.80	4.72±1.26	0.109
tridecanal	/	0.63±0.28	

Note: “/” indicates not be detected.

### Population growth of non-vector BPH on SRBSDV-infected and healthy plants

The population growth rate of non-vector BPH on SRBSDV-infected plants was 52.20±9.75, which had no obvious difference than its population growth rate of 57.75±2.28 on healthy plants (t = 0.501, df = 15.69, P = 0.623).

### Host selectivity and parasitic capability of *A. nilaparvatae* to BPH eggs on infected and healthy rice plants

**Figure 1:**
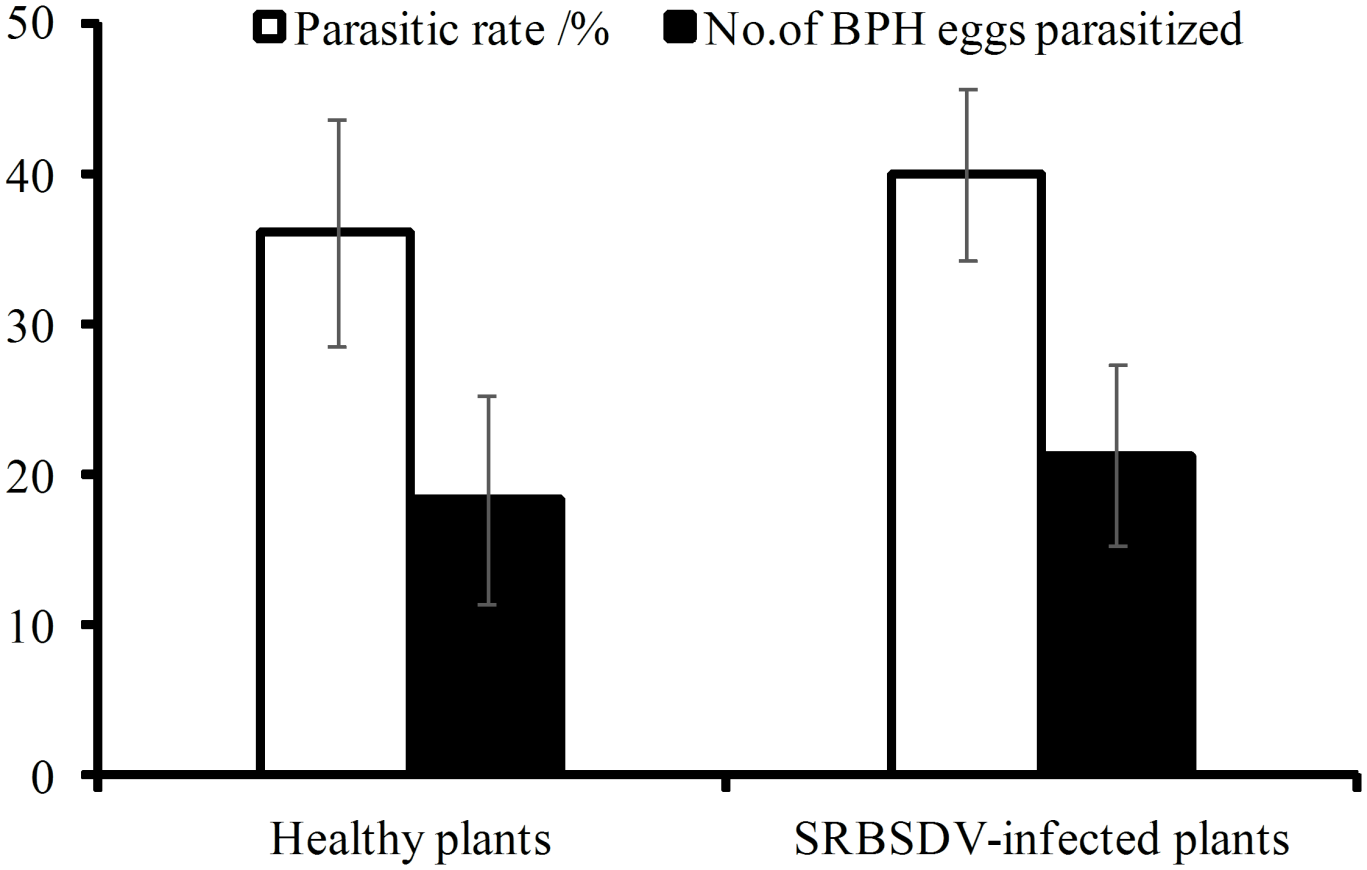
Effects of SRBSDV-infected rice plants on parasitic selectivity and parasitic capability of *Anagrus nilaparvatae*.

As shown in Figure 1, the parasitism rate of BPH eggs on SRBSDV-infected plants was 39.97%, whereas the parasitism rate on the corresponding healthy plants was 36.08%. The difference was not significant (t = 0.454, df = 14, P = 0.657).

Parasitic capability of *A. nilaparvatae* to BPH eggs on SRBSDV-infected plants was 21.32 per wasp, and that on healthy plants was 18.38. This difference was not significant (t = 0.941, df = 36.33, P = 0.353).

### Preference of *A. nilaparvatae* for odor sources from SRBSDV-infected and healthy plants

**Figure 2:**
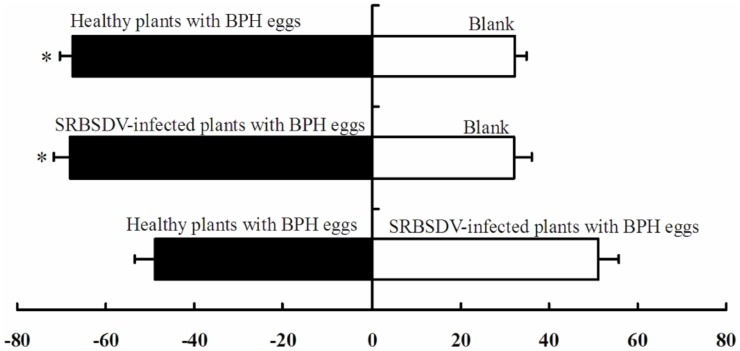
Preference of *Anagrus nilaparvatae* for different odor sources.

The results of preference testing of *A. nilaparvatae* on infected and healthy plants showed that *A. nilaparvatae* preferred rice plants with BPH eggs (t = 2.136, df = 4, P_healthy_ = 0.018; t = 2.771, df = 4, P_infected_ = 0.004). However, there was no significant preference for infected plants or healthy plants (P = 1.000) (Fig. 2).

### Behavioral responses of *A. nilaparvatae* to single volatile substances at different concentrations

**Figure 3:**
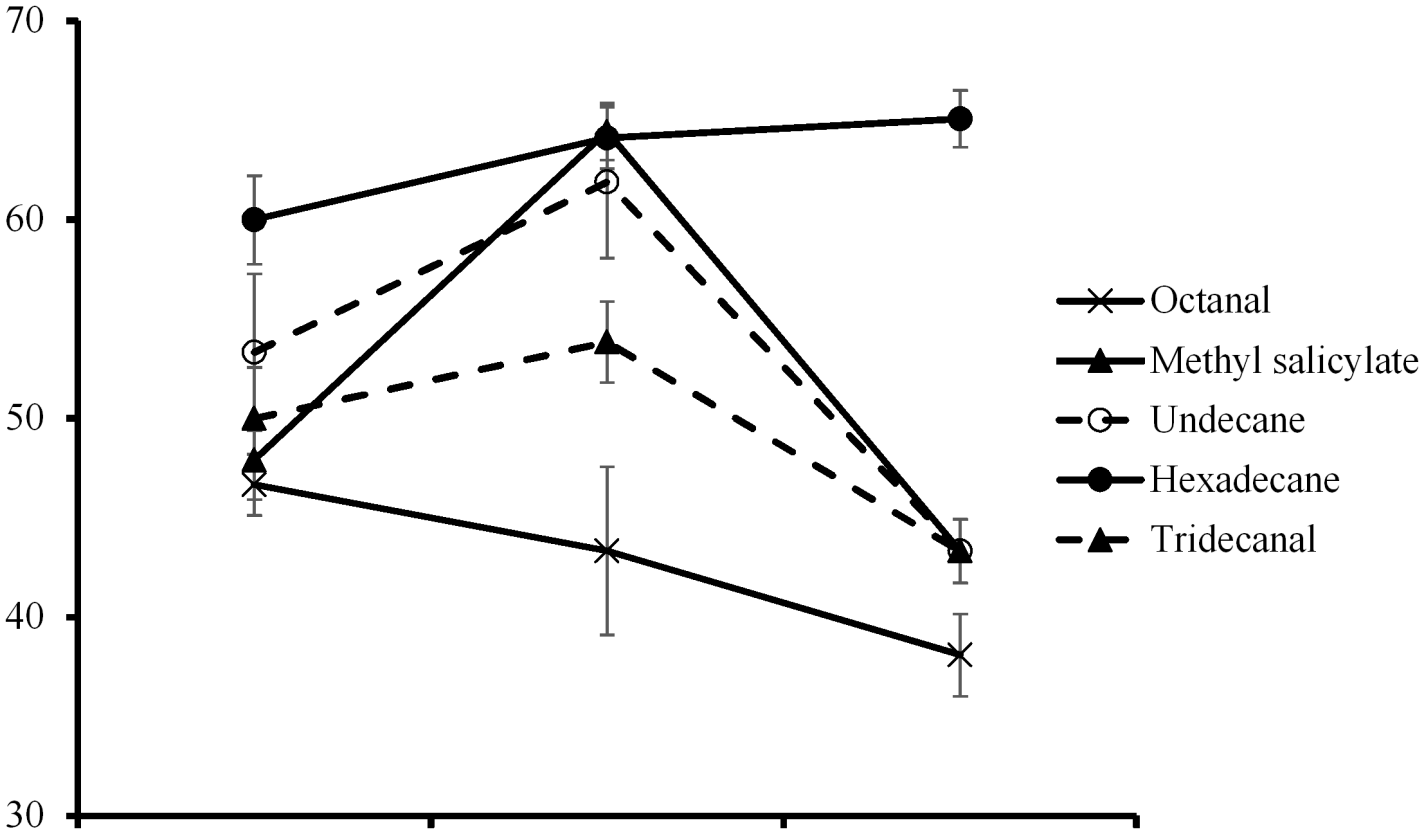
Behavioral responses of *Anagrus nilaparvatae* to single substance at different concentrations.

The results in figure 3 indicated that *A. nilaparvatae* had no obvious selectivity between single volatile substances at different concentrations and liquid paraffin in the control group. When the test substance was diluted 10^2^ times, *A. nilaparvatae* tended to choose hexadecane or methyl salicylate compared with the control group. When diluted 10^4^ times, four of the substances had a certain attractiveness for *A. nilaparvatae*. Octanal was an exception. When it was diluted10^6^ times, the proportions of *A. nilaparvatae* between the two odor sources were close to equal.

When pure products were diluted 10^2^–10^6^ times, methyl salicylate, undecane and tridecanal had the strongest attraction to *A. nilaparvatae*. With decreasing dilution, the attraction of octanal to *A. nilaparvatae* also decreased; the opposite was true for hexadecane.

## Discussion

Plant viruses can have positive [16], [30], [31] or negative [32], [33] impacts on the growth, survival and reproductive capability of herbivorous insects. For persistent viruses, the vector has a long time to acquire viruses, allowing viruses to induce changes in plant physiology and biochemistry that will affect vector insects. This is conducive to the spread of viruses [15], [34]. Our results showed that after SRBSDV infection, rice plants had no significant changes in amino acids and soluble sugar contents. Tu et al. (2013) introduced WBPH on SRBSDV-infected rice plants to feed for 48 h, and later transferred them to healthy plants. Their growth and development were then observed [22]. They found that, compared with WBPH feeding on healthy plants (16.8±0.4 d), the nymph duration of planthoppers feeding on SRBSDV-infected plants were prolonged (23.6±0.5 d) at 20°C, though there was no significant difference at 25 and 28°C. The duration of infection on SRBSDV-infected plants may have been responsible for these differences. Tu et al. (2013) showed the direct impact of a virus on vector insects, which was similar to our previous result that virus-carrying WBPH can significantly inhibit the reproductive ability of WBPH serving as the vector [22], [23]. In addition, SRBSDV infection could affect the feeding behavior of vector WBPH. Viruliferous WBPH fed in phloem more frequently than non-viruliferous WBPH, which would increase the probability of virus inoculation [23]. However, SRBSDV-infected plants had no significant impacts on the non-vector planthopper and its egg parasitoid, *A. nilaparvatae*, indicating no change happened in biological control by egg parasitoid of vector WBPH, since WBPH shares the same natural enemies with BPH [35]. Meanwhile WBPH should get more attention than BPH in the paddy fields of epidemic of SRBSDV.

There are few reports on the impact of plant viruses on non-vector insects. In a study of non-vector Q-type *Bemisia tabaci* feeding on tobacco infected with tomato yellow leaf curl china virus (TYLCCNV), the adult longevity and fecundity of *B. tabaci* were higher than those feeding on healthy tobacco [36]. Non-vector BPH and WBPH feeding on RBSDV-infected rice plants had improved ecological fitness, and this promoted the expansion of non-vector insect populations under natural conditions [5], [37]. However, Iris yellow spot virus (IYSV) had no significant impact on the survival of its non-vectors, *Frankliniella occidentalis*, *Frankliniella schultzei*, and *Frankliniella schultzei* [38]. Our present results showed that SRBSDV had no significant impact on the population growth of non-vector BPH. This finding can be explained by the fact that both amino acid and soluble sugar contents in rice plants did not change after SRBSDV infection. However, a previous study found that both amino acid and soluble sugar contents were significantly increased in the rice plants with RBSDV infection than in healthy plants, resulting in higher nymphal survival rate, female adult weight and egg hatchability of non-vector BPH, as well as its higher activities of defense enzymes and detoxifying enzymes [5].

Plant viruses can have indirect effects on natural enemies through vector insects. They can also have direct effects on natural enemies' growth and reproduction. For example, larval development of *Aphidius ervi* in *Sitobion avenae* was significantly delayed when barley yellow dwarf virus (BYDV) acquisition took place before or shortly after the parasitoid had hatched, but not when the parasitoid was at the second larval stage during virus acquisition. Similarly, the presence of BYDV led to significantly higher aphid mortality when they acquired virus up to and including the time that *A. ervi* was at the first larval stage. Adult female parasitoids deposited fewer eggs in viruliferous aphids [39]. Host selection by female parasitoids consists of host location, recognition, acceptance, as well as judgment of the suitability of the host for its development [40]. Our study showed that SRBSDV-infected plants have no significant impact on host selectivity and parasitic capability of *A. nilaparvatae*. To clarify differences in volatile secondary metabolites between SRBSDV-infected plants and healthy rice plants using GC-MS, the behavioral responses of *A. nilaparvatae* to octanal, undecane, methyl salicylate, hexadecane and tridecanal at different concentrations were tested. *A. nilaparvatae* exhibited no significant selectivity between single volatile substances at different concentrations and liquid paraffin in the control group. Rice volatiles are the major carriers in compound communication between rice plants, planthoppers and *A. nilaparvatae* [41]. It has been found that SRBSDV-infected plants are attractive to vector WBPH [42], whereas we found in this study that the egg parasitoid *A. nilaparvatae* has no significant selectivity to odor sources from infected and healthy plants, as well as no differences in behavior responses to single substances. A possible reason is that *A. nilaparvatae* is a parasitoid, and SRBSDV does not spread throughout the eggs. In comparison, the number and quality of eggs may have a larger impact. Among the three virus-mediated trophic levels of rice plants, planthoppers and parasitoid, SRBSDV-infected plants do not have obvious impacts on the non-vector BPH and its parasitoid.

With regard to spread of the virus, the number of vector insects is only one factor affecting the efficiency of spread. Through predation or parasitism, predators can also affect the spread of viral diseases [43]. When *Aphidius ervi* was introduced to the system of *Vicia faba*, pea enation mosaic virus (PEMV), and *Acyrthosiphon pisum*, the number of pea aphids, their longevity were reduced, which in turn lowered damage to host plants and plant susceptibility. Furthermore, *Acyrthosiphon pisum* parasitized by *Aphidius ervi* are more active, accelerating the spread of viral disease [43]. Introduction of the predator *Coccinella septempunctata* improves the infection rate of barley yellow dwarf virus (BYDV) spread by *Rhopalosiphum padi*, however, the parasitoid *Aphidius gifuensis* has no significant impact on the infection rate of healthy plants [44]. It is clear that spread of the virus is induced indirectly by predators in the system of predators, vector insects and host plants. Impacts of plant viruses on host plants, herbivorous insects, natural enemies and the entire ecosystem can be complex. Further study is needed to fully understand the interactions involved.
